# High Bonding Temperatures Greatly Improve Soy Adhesive Wet Strength

**DOI:** 10.3390/polym8110394

**Published:** 2016-11-08

**Authors:** Charles R. Frihart, Thomas Coolidge, Chera Mock, Eder Valle

**Affiliations:** 1 Forest Products Laboratory, Madison, WI 53726, USA; 2 AgriChemical Technologies, Mount Horeb, WI 53572, USA; thomas@agchemtech.com (T.C.); chera33@yahoo.com (C.M.); eder@agchemtech.com (E.V.)

**Keywords:** soy, flour, concentrate, isolate, wet shear strength, bonding temperature

## Abstract

Soy wood adhesive bond strengths reported in different literature studies are difficult to compare because a variety of temperatures and other conditions have been used for the bonding and testing step. Some reports have indicated bond strengths are sensitive to bonding temperature, but the reason(s) for this has not been intensively investigated. Although these prior studies differ in other ways (such as type of soy, wood species, and test method), the effect of bonding temperature has not been clearly examined, which is important for focusing commercial applications. A tensile shear test using two-parallel-ply veneer specimens with smooth maple was used to measure both the dry and wet cohesive strength of soy adhesives. Although the soy adhesives gave very good strengths and dry wood failure, they often have low wood failure and shear strengths under wet conditions when bonded at 120 °C. However, wet strength greatly increased as the bonding temperature increased (120, 150 and 180 °C) for these two-ply tests with. This study examined the use of different types of soys (flours, concentrates and isolates) and different bonding temperatures and bonding conditions to evacuate several possible mechanisms for this temperature sensitivity, with coalescence being the most likely.

## 1. Introduction

Soybean adhesives have been important to the wood bonding industry and led to the development of the interior plywood market because of their availability and good adhesive performance. These adhesives could either be cold pressed or hot pressed at 110–130 °C for more rapid curing [1]. Development of the natural gas and petroleum chemical industries, along with the great improvement in the performance of synthetic adhesives, led to a nearly complete replacement of soy adhesives by urea-formaldehyde (UF) adhesives in the 1960s. Hot-pressed UF adhesives cure rapidly at 120 °C by adding ammonium sulfate or ammonium chloride to the adhesive prior to application [2]. High temperatures are sometimes used with very low moisture content veneer, but warping can occur under these conditions. To minimize energy costs, surface veneer discoloration, and in some cases adhesive decomposition by over curing, the industry has recommended 120 °C as a bonding temperature for UF and phenol-formaldehyde adhesives (in agreement with Sedliačik et al. [3]). In some cases with UF adhesives, excessive bonding temperatures or bonding duration can lead to lower strength [4]. Despite the strength drop off at higher temperature, formaldehyde emissions were lower, making it the more favorable bonding condition.

Renewed interest in bio-based adhesives has led to a larger number of soy adhesive studies, using a wide range of methods to increase strength the adhesive strength [5] in ways different from the previous methodology of very high pH values and highly formulated soy adhesives [1]. Although these older formulations often used room temperature bonding, most current soy formulations do not bond well at room temperature. The question concerning the best temperature for bonding with soy near neutral pH values then arises. As evaluated by wet shear strength, Li et al. [6] found that the best temperature was 120–125 °C for making plywood bonds using poplar veneers and soy flour co-reacted with an epoxy and polyvinyl acetate. In contrast, some research has used up to 180 °C for bonding lap shear samples with various treatments of soy protein isolates [7]. Effect of heat treatment on soy products has been examined in a few cases [8,9], but it has not been broadly examined nor has the mechanism been examined in detail. Heating of soy protein isolate before bonding has been shown to increased bond strength [10,11].

To study the effect of bonding temperature, a variety of soy products were bonded at 120, 150 and 180 °C using an Automated Bond Evaluation System (ABES) bonder and tester; the ABES system uses small heated platens for bonding thin smooth veneers as lap shear specimens [12,13]. The strength of these samples was tested both dry and after a water soak, with emphasis on the latter because it is a more sensitive performance criteria [14,15]. With this method, we can address different mechanistic questions. Does the effect of bonding temperature apply to all flours independent of whether they are in the native state or heat denatured state? Does the same effect occur when the soluble carbohydrates, which are most likely to engage in Maillard reactions with the proteins, are removed as in the concentrates and isolates? Does the ethanol denaturation of the soy in removing carbohydrates to form a concentrate and jet cooking of the concentrate influence the temperature effect? Does the removal of almost all the carbohydrates in making the isolate change the effect of temperature on bonding? Is the high pressure necessary for good bond strength and does reheating a lower temperature bonded sample gave improved strength when reheated at a higher temperature?

## 2. Experimental

### 2.1. Materials

Hard maple rotary cut veneers of 0.6 mm thickness were obtained from either Columbia Forest Products (Greensboro, NC, USA) or Besse Forest Products Group (Gladstone, MI, USA) for the ABES testing. Soy products include Prolia™ 200-90, Prolia™ 200-70, Prolia™ 200-20 soy flour, (Cargill Inc., Cedar Rapids, IA, USA), and PRO-FAM™ 974 soy protein isolate, ARCON™ F and ARCON™ S soy concentrate (ADM, Decatur, IL, USA). Other chemicals used included 1.5 M NaOH and 1.5 M H_2_SO_4_ (Fisher Scientific LLC, Pittsburgh, PA, USA).

Lab soy protein isolate (LSPI) was isolated from Prolia™ 100-90 soy flour using pH to control protein solubility. Approximately 140 g of soy flour and 1400 g of water (~8% soy solids mixture) were mixed aggressively at pH 8 (using several additions of NaOH) to dissolve most of the proteins. The mixture was evenly distributed between six plastic centrifuge bottles for centrifuging at 10–15 °C with a 17 min centrifuge at 7750 rpm. Residual solids (mainly insoluble carbohydrates) were discarded and the supernatant returned to the mixing vessel after centrifugation. Adjustment of the supernatant with 1.5 M H_2_SO_4_ (~20 mL) to a target pH of 4.5 ± 0.05 was required to precipitate the protein. Centrifugation was then performed in the same manner as above. The solids were kept (isolated protein) and the supernatant (soluble carbohydrates) was discarded. Isolated protein was returned to the mixing vessel along with both water (800 mL) and 1.5 M NaOH (~40 mL). Full dispersion and neutralization of the protein at a pH of 7.0 ± 0.05 was achieved prior to lyophilization. Upon completion, the protein was ground into a powder and passed through a 100 mesh screen prior to storage.

### 2.2. Adhesive Preparation, Bonding and Testing

Soy dispersions were made by first measuring the appropriate amount of dry soy product into a beaker. Water was then added until the desired amount (15%, 20%, or 25% by solids) of soy was attained. The mixture was then hand stirred vigorously for 10 min with a glass stir rod, covered with Parafilm, and allowed to stand 1 h before restirring and testing. Water content of the soy solids was taken into account when making these dispersions.

The various soy dispersions were tested for their adhesive strength using an ABES from Adhesive Evaluations Systems, Inc. (Corvallis, OR, USA) to form bonds and to break the bonded samples for determining peak strength and wood failure. Veneers were cut into strips 117 mm long (along the grain) by 20 mm wide for test samples and equilibrated at 22 °C and 50% relative humidity (RH) for at least 24 h before cutting and bonding. The adhesive was quickly mixed (<30 s) before applying a 5-mm-wide strip of adhesive to one wood piece. Another wood piece was then immediately overlapped onto the adhesive-coated veneer and hot-pressed in the ABES unit at 0.25 MPa for 120 s at 120, 150, or 180 °C. All shear samples (10 for each combination) were allowed to equilibrate at 22 °C and 50 %RH at least overnight before testing. For testing, half the shear specimens for each combination were tested dry; the other half were tested after a 4 h water soak at 22 °C using the ABES unit to grip and then pull the samples until failure. The sample fracture often occurred in the wood both outside and inside the bonded area for dry samples, whereas failure was mostly in the adhesive layer for the wet samples.

To examine the effect of pressure and post-bonding heat treatment, a low pressure oven bonding method was developed. Oven bonding of shear samples involved only slight modification of the procedure described above, with the main differences being that only light pressure was applied to the bonded area and that the entire specimen was heated in an oven. Two 20-mm-wide steel strap strips were placed above and below the wood specimens to help distribute the force of the binder clips over the entire 20- by 5-mm binding overlap. Two binder clips, one on each side perpendicular to the bonded area, were used to keep the glueline in contact during curing. The clipped samples were placed in an oven to cure at 120 or 180 °C for 10 min. After heat curing, the samples were equilibrated overnight at 22 °C and 50 %RH, the same as the other shear samples. Reheating of bonded shear samples occurred after the overnight equilibration period. Whole shear samples to be reheating with ABES were simply placed in the grips, pressed at either 120 or 180 °C for 2 min, removed from the ABES unit, and then allowed to equilibrate overnight at 22 °C and 50 %RH. Reheating with the oven employed the use of binder clips and steel strapping in the same manner but on previously samples bonded at 120 °C. Shear samples were re-cured for 10 min at 120 or 180 °C and then allowed to equilibrate overnight at the same 22 °C and 50 %RH.

In an effort to better understand bond strength increase through greater heating, three-ply poplar veneers from Armstrong (Lancaster, PA, USA) were cut to be 152.4 by 152.4 mm and treated at bondline temperatures of 120, 150, and 180 °C. The veneers were conditioned at 27 °C and 30 %RH for at least 48 h. A Carver laboratory press model M (Wabash, IN, USA) was used for pressing panels, and the temperature of the bondline was recorded using the recommended thermocouple wire (36 gauge T-type duplex insulated thermocouple wire Omega Engineering (Stamford, CT, USA)). Temperature was monitored using a DATAQ Instruments model EL-USB-TC-LCD (Akron, OH, USA) data logger. For these tests, 5 g of a 24% solids 200-90 Prolia™ soy flour formulation (post 30 min mix) was used on each surface of the middle veneer cross-ply. All three-ply poplar panels were treated under the same conditions: 4 min open time for spreading the adhesive, no stand time, 5 min at 689 kPa of cold press, 5 min to reach target bondline temperature at 862 kPa, and 2 min at 120, 150, or 180 °C for hot press using thermocouple to verify bondline temperature. The completed boards were conditioned at 32 °C and 20 %RH overnight before cutting into specimens for ASTM D906-98(2011). A Brookfield LVF Viscometer (Brookfield AMETEK, Middleboro, MA, USA) was used to measure the viscosity of the resin at a set rotation per minute (RPM) using spindle #3.The qualitative analysis also included measuring the pH of our resin.

## 3. Results

### 3.1. Soy Flour

Given the natural variability of wood, care needs to be taken to observe reliable differences. This led to the use of smooth and defect-free surface veneers of hard maple for testing. The ABES small-scale bonding and strength testing equipment was developed to understand the kinetics of strength development during curing for modelling adhesive bond strength development during composite production. This process has been established as method ASTM D 7998-15. Thus, this equipment was useful for testing the effect of temperature on bond strength with a variety of soy products. Additionally, we previously investigated the comparability of the tests against normal scale tensile shear specimen and found good correlation [14]. In testing wood adhesives, dry adhesive strengths are often greater than wood strength; this changes dramatically with water exposure because the adhesive usually fails before the wood. Although both dry and wet tensile shear strengths from the ABES test were obtained, wet testing results are emphasized because the differences in adhesive failure are more informative. Some key samples were repeated to demonstrate the reproducibility of the method.

**Figure 1:**
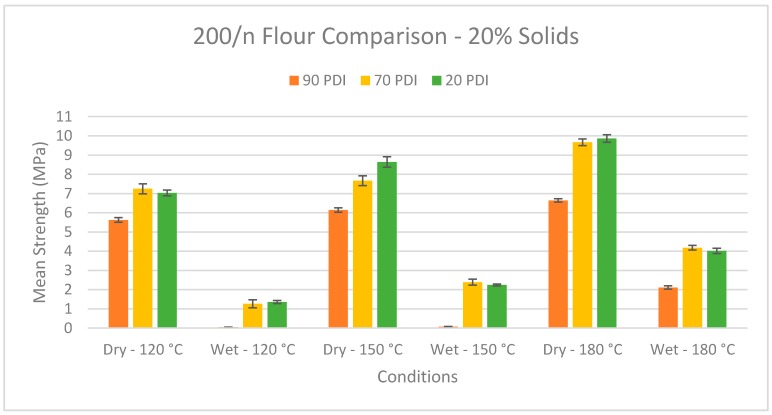
Shear strengths of soy flour adhesives with maple veneer bonded at different temperatures and tested dry and wet.

Our prior work showed that soy flour provided a conclusion contrary to the common assumption that the more dispersible the protein was, the better the bond strength should be with ABES testing [15]. The data here also support this prior observation because the native flour with the highest protein dispersability index (PDI), the 90-PDI flour, showed the lowest dry and wet strengths. However, the strength of all soy flours tested increased in wet strength with increase in the bonding temperature from 120 to 150 to 180 °C. Interestingly, the 90-PDI flour never caught up to the other two flours (Figure 1). The effect of bonding temperature changes exists within all three main types of flour: the native 90-PDI, 70 PDI, and most denatured 20-PDI.

### 3.2. Soy Concentrate

One theory is that the higher temperature produces more Maillard reactions, normally characterized as the browning reaction between carbohydrates and proteins [16], creating higher bond strength [17]. Soy flour with about 45% protein and 45% carbohydrate makes Maillard reactions seem reasonable. For this reaction, the carbohydrate needs to be a reducing sugar, which has an aldehyde for reacting with the amine on the protein. The Maillard reaction is a series of reactions between proteins and carbohydrates that can generate higher molecular weight products and brownish color [16].

**Figure 2:**
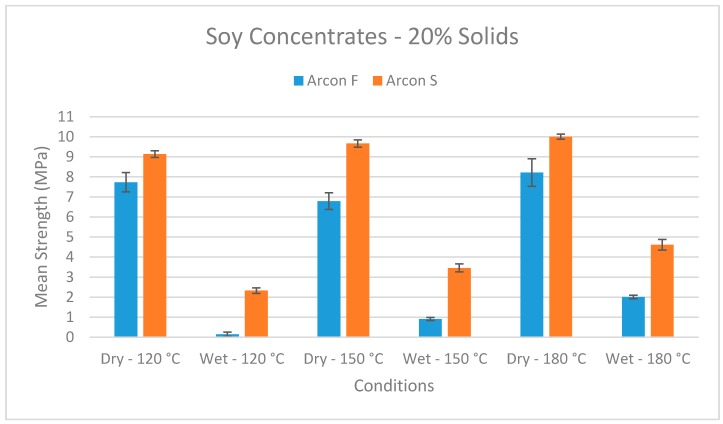
Wet shear strengths of soy concentrate adhesive with maple veneer bonded at different temperatures.

Most of the reducing sugars in soy flour are soluble carbohydrates, given their composition of sucrose, raffinose, and highers [18,19]. One way to test if the Maillard reaction is an important contributor to higher wet strength is to use the concentrates with most of the reducing sugars removed so that there are fewer reactive carbohydrates. Concentrates (about 75% protein and 20% carbohydrates) are made from flour that has been extracted with aqueous ethanol to remove soluble carbohydrates and low molecular weight proteins. Having fewer reactive carbohydrates should reduce the effect of any possible Maillard reactions, and thus greatly reducing the enhancement of wet bond strength by higher bonding temperatures compared to the flour [17]. However, this was not observed for two commercial concentrates (Figure 2). The higher bonding temperatures were very effective in improving wet bond strength. The two concentrates differ in that the Arcon S concentrate was jet cooked and the Arcon F concentrate was not. Although jet cooking improved wet strength of the concentrate, the higher bonding temperature provided additional strength under wet conditions.

### 3.3. Soy Protein Isolate

To further test the evaluation that improved wet strength is not due to protein–carbohydrate reactions, soy protein isolate with over 95% protein content (SPI) was tested. This research has been done on different types of SPI [20,21]. Producing laboratory SPI (LSPI) involves separating the protein from both soluble and insoluble carbohydrates in a mild manner to yield what is normally considered the native state. The procedure detailing LSPI production is given in the experimental section; we found this procedure to be better than others in the literature for adhesive strength [20,21]. Furthermore, the commonly used SPIs are available commercially (CSPI) and follow a somewhat similar production procedure as the LSPI, but they are jet cooked to give better functionality in food applications. This also greatly increases their viscosities [17,22]. Both of these SPIs contain only a few percent of carbohydrates. Because of the great difference in viscosity, LSPI is generally used at a greater solids level than CSPI to provide consistent bond strengths. Above 15% solids, CSPI becomes too viscous to spread, and below 10% LSPI is too thin to develop a good bond.

**Figure 3:**
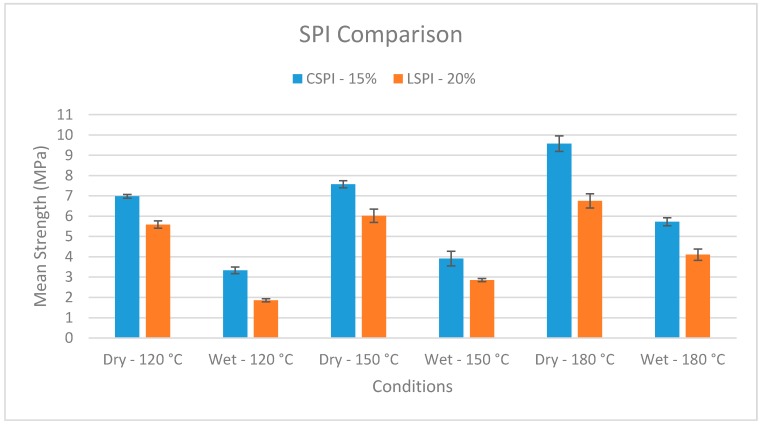
Wet shear strengths of soy protein isolate adhesive with maple veneer bonded at different temperatures. CSPI: commercially available soy protein isolate; LSPI: lab soy protein isolate.

High temperature bonding of both LSPI and CSPI gave improved strengths. At 120 °C, the LSPI is lower in wet strength than the CSPI. Both increase in strength at 150 °C, and then again at 180 °C (Figure 3). Given the higher initial strength of the LSPI and CSPI, the increase is less dramatic than on the lower strength soy flour adhesives, which have low strengths at 120 °C, Data for LSPI are supported by another study that used a somewhat different LSPI isolation, bonding, and testing process [23,24]. As with the concentrate, the jet-cooked material [22] possesses a higher strength than its non-jet-cooked counterpart. Additionally, the increase with jet cooking is maintained despite the increase in strength seen with higher bonding temperatures.

### 3.4. Oven Bonding and Reheating

The results with soy concentrate and isolate added more doubt concerning the postulate that increased strength is a function of carbohydrate–protein reaction. The data are more supportive of protein–protein interactions.

The question arises as to whether it is critical for the higher temperature to be in the initial bonding process when the aqueous protein is more mobile than the dried adhesive or whether simply higher temperature exposure in general is important for improved water resistance. This was evaluated by bonding samples initially at 120 °C and then later exposing the samples to 180 °C temperature using either the ABES unit with normal bonding pressure or in an oven with mild clamping to minimize sample distortion. Table 1 lists the specific reheating and bonding conditions for both the CSPI and LSPI samples.

**Figure 4:**
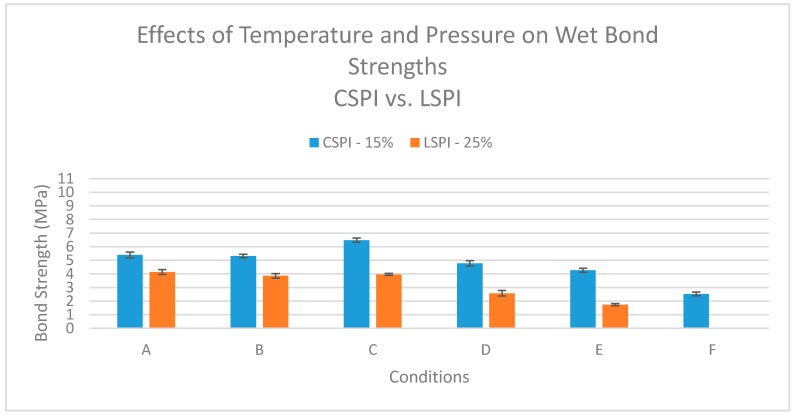
Wet shear strengths of CSPI and LSPI adhesive with maple veneer bonded under different conditions (defined in Table 1).

For the samples bonded in the ABES at 120 °C, heating them to 180 °C in ABES under pressure (A) or in an oven (B) gave similar wet strength shear tests as bonding at 180 °C in the ABES for the LSPI and close to the same strength for CSPI (C) (Figure 4). Thus, heating to the higher temperature, rather than the dynamics of the initial bonding process, is critical for higher strength. However, the pressure of bonding helps either with the transfer or the consolidation of the adhesive because bonding at 180 °C is not quite as effective when only light pressure is on the bondline in the oven (D) compared to ABES bonding (C). Case E in Figure 4 shows that bonding at 120 °C for 10 min in the ABES does give some improvement in bond strength nearly matching the higher temperature strength values seen with normal 2 min bonding as shown in Figure 3. Case F in Figure 4 shows that bonding under light pressure in the oven at 120 °C was not very effective for the LSPI, probably due to its poor transfer with a lower viscosity, but was somewhat effective for the thicker CSPI.

### 3.5. Plywood Bonding

Although the ABES shear data are very valuable for identifying the important factors for generating bond strength, the actual application is in bonding plywood. Thus, we needed to test the effect of temperature on plywood bond strength. CSPI was used as the adhesive in this set of three-layer plywood experiments. Using three-layer plywood, conditions had to be established for setting platen temperature to reach the desired bondline temperature and determining the time to reach this temperature. The latter was from 5 to a bit over 6 min for 120, 150, and 174 °C. The bondline temperature for 180°C could not be reached without scorching the poplar plywood; instead 174 °C can be used without scorching the wood.

**Figure 5:**
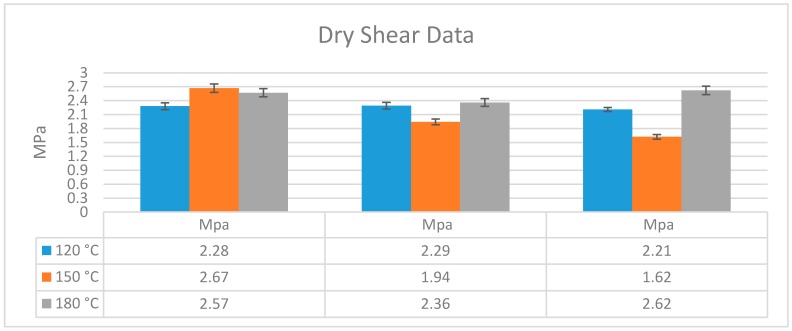

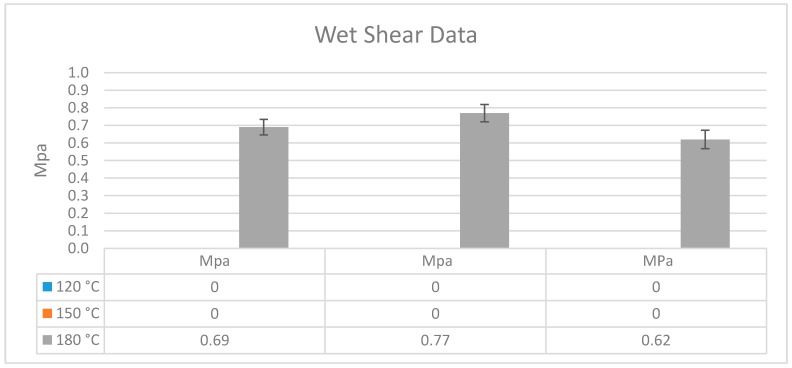
Dry and wet shear strength for three samples of CSPI bonded three-ply plywood.

For each of the three plywood assemblies, four samples were tested dry and four wet with checks pulled closed, otherwise using ASTM D906-98(2011). While there is little difference in the dry shear strengths, all shear specimens bonded at 120 and 150 °C failed as soon as they were removed from the 24 h soaking. At 174 °C, the shears were able to be tested and yielded an average of 0.69 MPa (Figure 5). The improvement in wet strength at 174 °C, compared to 120 and 150 °C, is probably due to protein coalescence to increase the ability of the treated resin to expand as the three-ply poplar veneer swelled.

## 4. Discussion

Various rationalizations have been provided in the literature with some support from microscopic and spectroscopic data. However, this additional data has been far from conclusive, given the lack of model compounds and highly specific techniques to measure protein tertiary and quaternary structures in a mixed protein-carbohydrate composition. Thus, we have taken an approach that actually measures the effect on adhesive strength under different temperature conditions with different soy materials so as to evaluate these rationalizations. The bond failure under wet conditions is cohesive within the adhesive; thus it is measuring the strength of the wet soy film.

One curiosity is why the less dispersible soy flours give better strength the 90 PDI flour. The prior treatment in making the 20 and 70 PDI soy seems to have caused greater association of the proteins that not only reduces their dispersibility, but also increases their cohesive strength. Thus, models that discuss certain treatment of the soy to give better adhesion the wood do not seem important when the 90 PDI flour that is more likely to provide good adhesion actually gave worse results.

One common theory hypothesizes that treatment of soy brings non-polar groups to the surface so that the protein is less sensitive to water. It is difficult to determine how this dramatic of a change of protein structure could occur in the experiments re-heating of the dry already bonded samples. In our studies, we were unable to see a difference in contact angles with water drops on soy coatings cured at 120, 150, and 180 °C. We also did not see appreciable differences in extractives for cured soy flours at the three temperatures. None of this information is reported here since we continue to look for more sensitive methods to try to detect if any real differences exist.

Especially for the soy flours, chemical reactions between the proteins and carbohydrates have been proposed for curing of the soy. The increased strength with heating for soy concentrate and isolate make it doubtful about the validity of the postulate that increased strength is a function of carbohydrate–protein reaction (Maillard-type reactions). The data are more supportive of protein–protein interactions.

Not much has been discussed in the literature about the coalescence between portion globules for improved bond strength. Localized distortion between the globules with increased temperature could provide better association of the proteins [25]. The interesting aspect of the data is that each 30 °C increase brought about 1 MPa increase in strength for each of the soy materials. This data could indicate that the additional heat is just making the aggregates larger by coalescence, which in turn provides the greater strength.

## 5. Conclusions

All the soy flours, concentrates, and protein isolates tested developed greater wet shear bond strengths at 180 °C than 150 °C and finally 120 °C in small-scale bonding tests. Each case provided about a 1 MPa increase for every 30 °C increase. This temperature difference can be mainly overcome by increasing the 2 min bonding conditions to 10 min to give similar bonding strengths. The difference is not a phenomenon of just initial bonding temperature with a highly fluid adhesive because reheating specimens at 180 °C that were bonded at 120 °C gave strength values similar to those of the 180 °C bonded specimens. Transfer of adhesive to the other surface seems to be an important issue since oven bonding under light pressure was not as effective as bonding under pressure in the ABES. The previously proposed Maillard reaction does not seem to play a role since the isolates and concentrates increase wet strength as much as the flours even though they do not contain the necessary reducing sugars.

The best explanation for the strength increase seems to be the coalescence of the protein globules caused by minor rearrangement of the protein at the higher temperature. Limited internal unfolding and refolding could allow greater interactions between protein chains without necessarily disturbing the preexisting strong interactions already in commercial soy protein isolate and concentrates that have been jet cooked.

## Figures and Tables

**Table 1:** Reheating and oven bonding conditions for bonding under different conditions for both the CSPI and LSPI. CSPI: commercially available soy protein isolate; LSPI: lab soy protein isolate; ABES: Automated Bonding Evaluation System.

Conditions	
A	Bonded @ 120 °C with ABES, Reheated @ 180 °C with ABES
B	Bonded @ 120 °C with ABES, Reheated @ 180 °C in oven (10 min)
C	Bonded @ 180 °C with ABES
D	Bonded @ 180 °C in oven (10 min)
E	Bonded @ 120 °C with ABES (10 min)
F	Bonded @ 120 °C in oven (10 min)
